# Role of High-Sensitivity C-reactive Protein (Hs-CRP) in Non-communicable Diseases: A Review

**DOI:** 10.7759/cureus.30225

**Published:** 2022-10-12

**Authors:** Tanvi Banait, Anil Wanjari, Vedika Danade, Shashank Banait, Jyoti Jain

**Affiliations:** 1 Internal Medicine, Jawaharlal Nehru Medical College, Datta Meghe Institute of Medical Sciences, Wardha, IND; 2 Internal Medicine, Mahatma Gandhi Institute of Medical Sciences, Wardha, IND; 3 Ophthalmology, Jawaharlal Nehru Medical College, Datta Meghe Institute of Medical Sciences, Wardha, IND

**Keywords:** molecular biomarker, inflammation, high sensitivity c-reactive protein, c-reactive protein, acute phase reactants

## Abstract

Non-communicable diseases like cardiovascular diseases, cerebrovascular diseases, diabetes mellitus, and cancer are very common causes of death worldwide. Therefore, the need to search for novel, affordable, and easily accessible biomarkers and risk factors for non-communicable diseases continues, which can predict the future risk of having these diseases with greater accuracy and precision. In this context, among available biomarkers, high-sensitivity C-reactive protein (Hs-CRP) is considered to be the best-suited marker. Various drug intervention trials demonstrated positive results in reducing Hs-CRP in individuals with raised levels. Numerous pharmacological and non-pharmacologic interventions in the form of lifestyle modifications, exercise, and cessation of smoking are being investigated to study their effect on reducing serum C-reactive protein (CRP) levels.

This review article discusses the role of Hs-CRP and its isoforms in the pathogenesis of various disease conditions, factors affecting its serum concentration, its prognostic value, and its comparison with other risk factors. Further, its clinical significance in chronic inflammatory and degenerative diseases of the nervous system and other common non-communicable diseases, including recent advances in the management of various diseases, has also been discussed.

## Introduction and background

C-reactive protein (CRP), a positive acute phase reactant (protein), was first discovered in response to inflammation in the hepatocytes and released into the blood. It is an indicator of the ongoing inflammatory process and various diseases in our bodies. It was discovered from the serum of patients suffering from pneumonia caused by pneumococcal bacteria during the acute phase [1]. Although the term acute phase reactants is used, its level also increases in various chronic disorders like autoimmune diseases, malignancy, chronic wounds after injury, inflammatory conditions, and metabolic disorders [2].

It is commonly but incorrectly believed that high-sensitivity and conventional CRP are two different entities. The high-sensitivity C-reactive protein (Hs-CRP) is a biochemical test which is a highly sensitive quantification of CRP. High sensitivity is a new modified assay which measures very low levels of CRP in plasma. It gives us an estimate of general levels of inflammation in our body, giving an idea of the inflammatory status. The Hs-CRP is used as a predictive marker of cardiac disease risk and stroke in otherwise apparently healthy people or individuals with or without risk factors for the development and progression of these disease conditions [3]. The Hs-CRP test measures even low levels of inflammation and indicates the risk of cardiac disease and stroke [4].

## Review

Method

All relevant articles were identified and were systematically searched through the PubMed, Medical Literature Analysis and Retrieval System Online (MEDLINE) database, and UpToDate till June 2022 by using the following Medical Subject Headings (MESH) words: Acute phase reactants, autoimmune diseases, biomarker, cardiovascular diseases, cancer, c-reactive protein, cerebrovascular diseases, high sensitivity c-reactive protein, diabetes mellitus, inflammation, obstructive sleep apnea, and stroke. Our investigation included studies on humans of 18 years and above, published in the last 15 years, and in the English language, and studies performed on animals and in non-English language were excluded. Our study was primarily targeted to find the role of CRP in non-communicable diseases. We have reviewed selected references and cross-references of these articles and various textbooks also.

Discussion

After the analysis of selected articles, we found that a wide range of non-communicable diseases like cardiovascular diseases, diabetes mellitus, stroke, hypertension, cancer, obstructive sleep apnea, rheumatoid arthritis, age-related macular degeneration, Parkinson's disease, and Alzheimer’s disease are associated with CRP. This review article also discusses metabolism, biological functions, and various therapeutic options for CRP reduction.

Synthesis and metabolism

C-reactive protein, an acute phase reactant, belongs to the pentraxin family and is a key protein of innate immunity. It is produced mainly by the hepatic cells in response to increases in proinflammatory cytokines levels; during the transcription phase, interleukin (IL)-6, by its key transcription factors, upregulates the production of cytosine-cytosine-adenosine-adenosine-thymidine (CCAAT)-enhancer-binding proteins (C/EBP) β and C/EBPδ and regulates the synthesis of CRP in this process [5] IL-6 signaling may be additionally reinforced by tumor necrosis factor (TNF) and IL-1β, as both of these cytokines enhance the transcription rate of CRP [6]. Visceral adipose tissue also releases proinflammatory cytokines during the inflammatory process and contributes to raising serum CRP levels by increasing signaling [7]. Both raised levels of serum leptin and decreased adiponectin levels have a common association with obesity and insulin resistance. Both of these adipokine disturbances are related to increased synthesis of CRP from the liver [8,9], as well as hyperleptinemia is also associated with increased production of CRP in the cells of vascular endothelium [10].

In this setting, adipose tissue, being an endocrinal tissue, acts in an important immunomodulatory role. Adipose tissue releases inflammatory pleiotropic cytokines like IL-6, IL-1β, and resistin [11], which gives us an idea that chronic inflammatory status may be one of the basic components of obesity [12].

With increasing storage of the lipid in the adipocyte, there occurs hypertrophy, hyperplasia, and dysfunction of adipocytes, which is the main phenomenon in the pathogenesis of obesity [13].

These structural and metabolic changes in the adipocyte make them vulnerable to hypoxic injury and more prone to rupture easily, which ultimately results in the secretion of proinflammatory adipokine in adipose tissue [13,14]. It has been found that in response to various proinflammatory mediators, CRP is deemed to be expressed in lipocytes. This expression of CRP indicates a link between chronic inflammation and obesity [15].

A significant increase in levels of CRP in serum occurs at six-eight hours after the beginning of the inflammatory process, which initiates synthesis and then is released into circulation and peak levels are found between 24-48 hours, as its half-life is about 19 hours. Synthesis rate primarily determines CRP concentration in circulation [16]. Extrahepatic sites of CRP production occur as its messenger ribonucleic acid (mRNA) has been found in lymphocytes, lungs [17], adipose tissue [18], tubular epithelial cells in the renal cortex [19], and cells of smooth muscle and macrophages of atherosclerotic plaques [20-22]. Study conducted in the past showed that the extrahepatic origin of CRP production also contributes to CRP levels. This local CRP levels also help to predict the risk of the development of cardiovascular diseases, which includes evidence of CRP production in cells of smooth muscle in coronary arteries in response to inflammatory cytokines. Endothelial cell activation, which is produced by local CRP, has an important role in the development of various non-communicable disease conditions [22].

Serum CRP levels increase in various inflammatory conditions like acute infections, injury, and trauma. Along with the increase in CRP levels, there occurs an elevation of erythrocyte sedimentation rates (ESR) [23]. CRP is a direct measure of inflammation while ESR is an indirect measure and can be affected by conditions or factors unrelated to acute or chronic inflammation like increased age, female sex [24], anemia, end-stage renal disease [25] and obesity [26].

Steady molecular structure imparts CRP a long half-life (18 to 20 hours). Moreover, CRP levels remain stable as they have no diurnal variations and are not associated with food intake. Latest methods like highly sensitive enzyme-linked immunosorbent assay, immunoturbidimetry, immuno-nephelometry, and other modified assays are useful techniques that can detect serum CRP levels as low as 0.01 mg/dl to the range of 10 mg/dl [27]. High levels of Hs-CRP can be useful in detecting various low grades of systemic inflammatory disorders in the body without overt evidence of immunologic disorders or systemic inflammation. With the advancement of the Hs-CRP assay techniques, the levels can be measured accurately even from frozen plasma or fresh plasma [28]. The Hs-CRP is a perfect biological marker for cerebrovascular and cardiovascular risk prediction [29].

Biological functions of CRP

Native CRP (nCRP) is produced in response to infection or inflammation and it gets dissociated into five monomeric molecules (mCRP) irreversibly at the sites of inflammation. Apart from inflammatory processes, it has crucial roles in host responses to infection via activation of complement pathway, production of cytokines, like interleukin-6 and tumor necrosis factor-α., apoptosis, release of nitric oxide (NO), and phagocytosis. The two isoforms of CRP are different in configuration, molecular mass, antigenic properties, binding properties to different types of Fc gamma receptor and thus distinguished biological functions. The nCRP activates the classical complement pathway, promotes apoptosis, induction of phagocytosis, suppresses adherence of platelets to neutrophils, and have more anti-inflammatory properties compared to mCRP. On the other hand, mCRP can delay apoptosis by promoting the chemotaxis and recruitment of circulating leukocytes to areas of inflammation [30].

The Hs-CRP causes increased mobilization of monocytes into atheromatous plaque, which leads to suppression of basal and induced nitric oxide release from vascular endothelium, which finally causes endothelial dysfunction. The Hs-CRP have been found to increase the expression of plasminogen activator inhibitor-1 and other adhesion molecules in human coronary artery endothelial cells and systemic vascular endothelial. The Hs-CRP also enhances low-density lipoprotein-cholesterol (LDL-C) uptake by macrophages [31]; Hs-CRP directly contributes to the atherosclerotic process in vessels [32].

Hs-CRP in various disease conditions

Pathophysiology of Atherosclerosis and Role of Hs-CRP

In all phases of atherosclerosis, inflammation and its biomarkers have an important role that is from the early asymptomatic state (recruitment of circulating leukocytes) in the culprit blood vessel to the rupture of unstable vessel plaques leading to angina and myocardial infarction [33]. Numerous biomarkers of inflammation play a role in atherosclerosis, but CRP is the major one.

It is an independent and significant risk factor for the development of ischemic cardiovascular diseases [5-7]. Risk of development of ischemic cardiovascular diseases at various serum CRP levels [34] is shown in Table 1.

**Table 1:** Assessment of risk of cardiovascular disease based on serum Hs-CRP level.

Risk of cardiovascular disease	Hs-CRP level
Low	Less than 1 mg/ Litre
Average	Between 1-2 mg/ Litre
High	More than 2 mg/ Litre

**Figure 1:**
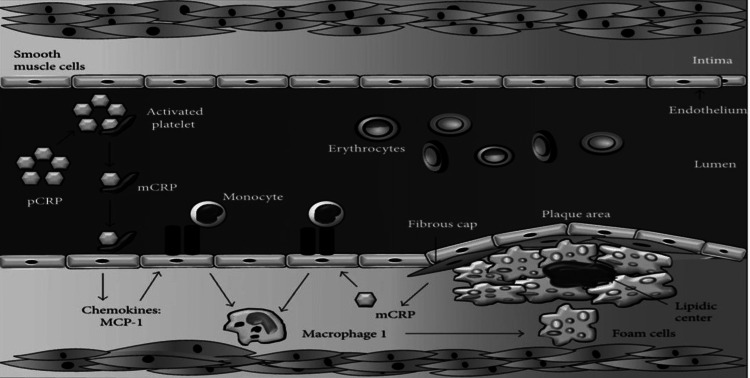
Role of Hs-CRP in the pathophysiology of atherosclerosis. MCP-1: Monocyte chemoattractant protein-1, mCRP: monomeric C - reactive protein, pCRP: pentameric C - reactive protein. Image source: Salazar et al., 2014 [37] (Open access)

It plays a vital role in the development of atherogenesis by various mechanisms like vascular cell activation, activation of the complement system, accumulation of monocyte, apoptosis, collection of lipids, and finally thrombosis in coronaries. The two isoforms of CRP are involved in the process of atherosclerosis: the pentameric CRP (pCRP) isoform attaches to the phosphatidylcholine present on the surface of apoptotic cells and starts inflammation [35], while monomeric (mCRP) modulates platelet function and promotes thrombosis inside the lumen of the vessels by stimulating platelet aggregation (Figure 1) [36,37].

Cardiovascular Disease 

**Figure 2:**
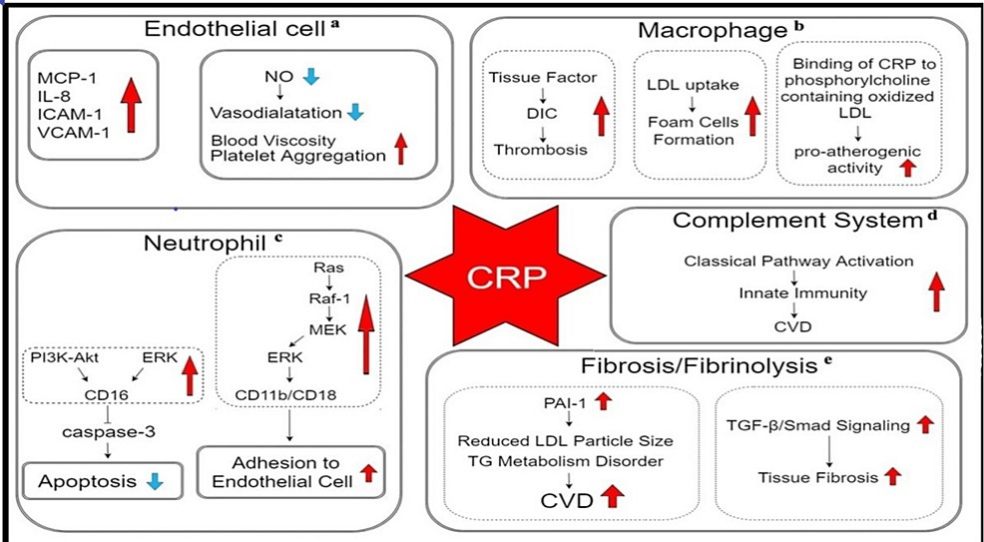
Role of CRP in the pathogenesis of cardiovascular diseases. CRP: C - reactive protein, CVD: Cardiovascular diseases, DIC: Disseminated intravascular coagulation, ERK: Extracellular signal-regulated kinase, ICAM-1: Intercellular adhesion molecule, IL-8: Interleukin-8, LDL: MCP-1: Monocyte chemoattractant protein-1, MEK: Mitogen-activated protein kinase kinase, NO: Nitrous oxide, PAI-1: Plasminogen activator inhibitor-1, PI3K-Akt: Phosphatidylinositol-3-kinase- Protein kinase B, Raf-1: Serine/threonine kinase, Ras: GTPase protein, TG: Triglycerides, TGF-β: Transforming growth factor-β,VCAM-1: Vascular cell adhesion molecule. Image source: Luan and Yao, 2018 [40] (Open access)

Patients with raised basal levels of CRP are at an increased risk of cardiovascular disease, type 2 diabetes mellitus, and systemic hypertension. The elevated CRP levels can increase the area of ischemic necrosis via complement activation leading to severe disease [38]. Therefore, the inhibition of CRP can be an innovative, effective, and safe therapy for the management of both myocardial and cerebral ischemia and infarcts. Arterial damage occurs due to the invasion of the leukocytes and other inflammatory processes within the vessel wall during ischemia. C-reactive protein is a common marker for both acute infection as well as inflammation, so it can be used as a marker of cardiovascular disease risk, although it is not a very specific prognostic indicator (Figure 2) [39,40].

The normal range of Hs-CRP is up to 1.0 mg/L. Levels more than this are found to be associated with more risk of cardiovascular disease. Levels more than 2.0 mg/L are associated with poor prognosis, higher rates of complications, and death [34,39]. To predict the development of future cardiovascular disease, Hs-CRP should be combined with other atherogenic molecules such as LDL-C, elevated levels of total cholesterol, low levels of high-density lipoproteins, increased triglycerides, and raised blood glucose (Table 2) [3,39].

**Table 2:** Results of various studies showing the role of Hs-CRP in CVD. Hs-CRP: High-sensitivity C-reactive protein, CVD: Cardiovascular diseases.

Sr. No	Author/ Year [Reference]	Type of Study	Conclusion
1	Kandelouei et al (2022) [41]	Systematic review and meta-analysis	This systematic review and meta-analysis showed that the statins are efficacious in reducing the concentrations of CRP and Hs-CRP in patients with different types of cardiovascular diseases and coronary artery diseases like acute coronary syndrome, myocardial infarction, stable atherosclerotic plaques, and unstable angina.
2	Gholoobi et al (2021) [42]	Randomized, placebo-controlled, double-blind clinical trial.	Hs-CRP is an important inflammatory marker for non-ST-segment elevation myocardial infarction and is related to cardiovascular events.
3	Carrero et al (2019) [34]	Randomized controlled trial	Most patients with myocardial infarction exhibit elevated Hs-CRP levels and Hs-CRP ≥2 mg/L levels were associated with a higher risk of major adverse cardiovascular events (adjusted hazard ratio, 1.28) and death (adjusted hazard ratio, 1.42). Increased Hs-CRP levels have prognostic validity as biomarkers beyond the trial evidence; in real‐world healthcare-based settings.
4	Tayefi et al (2017) [43]	Decision tree algorithm	This study indicated that serum Hs-CRP levels are the most important variable associated with coronary artery diseases and this biomarker has a stronger association with coronary heart disease in comparison to traditional biomarkers such as fasting blood glucose and low-density lipoproteins.
5	Li et al (2017) [44]	Meta-analysis	The risk of cardiovascular mortality increased by 2.03 times among study subjects with the highest Hs-CRP levels. Hs-CRP can stratify all-cause and cardiovascular mortality risks in the general population.
6	Wang et al (2017) [45]	Cross-sectional	This study concluded that cumulative exposure to Hs‐CRP was associated with a subsequent increased risk of CVD and myocardial infarction in a dose-dependent manner. Cumulative measurements of Hs-CRP are better than single measurement of risk prediction of disease development of CVD and myocardial infarction.

Stroke and Hypertension

Stroke is one of the leading causes of death and long-term disability [46]. Many patients with stroke with increased levels of CRP have a greater risk of death within 72 hours of stroke event in comparison to patients having normal CRP levels. There is much evidence that CRP increases in inflammatory diseases and atherosclerotic diseases. Hs-CRP, being more sensitive to vascular inflammation, can be used to prognosticate, to judge the extent of brain necrosis as a marker for vascular events. Raised levels of serum Hs-CRP are found to be strongly associated with an increased risk of stroke; therefore, Hs-CRP levels are used for the primary prevention of stroke in individuals with other risk factors for stroke and initiate preventive therapy (Table 3) [47].

**Table 3:** Results of various studies showing the role of Hs-CRP in stroke.

Sr. No	Author/Year [Reference]	Type of Study	Conclusion
1	Wang et al (2021) [48]	Case-control	The results of this study revealed that elevated Hs-CRP (inflammatory marker) levels along with multiple acute infarctions (imaging marker) are efficient markers for one-year stroke risk stratification in patients with minor ischemic stroke or transient ischemic attack compared with using them alone.
2	Alikiaii et al (2021) [49]	Systematic review	The results of this study suggest that statin therapy has beneficial impact in patients after a stroke by reducing CRP levels. Statin therapy should be considered in these patients with stroke, irrespective of their levels of cholesterol as potential anti-inflammatory agents.
3	Kitagawa et al (2017) [50]	Randomized controlled trial	During follow-up, Hs-CRP levels are good predictors of vascular events and recurrent stroke among patients of ischemic stroke. Pravastatin therapy may reduce vascular inflammation as assessed by Hs-CRP levels in study subjects with non-cardiogenic ischemic stroke.
4	Zhou et al. (2016) [51]	Meta- analysis	This meta-analysis reported that patients with elevated level of Hs-CRP had 46% excessive risk of ischemic stroke but slight reduction in risk of development of hemorrhagic stroke. The association of Hs-CRP level and risk of ischemic stroke was more pronounced in men than in women.
5	Jiménez et al (2015) [52]	Cross-sectional	This study concluded that even after adjustment for various cardiovascular risk factors and potential confounders; raised levels of Hs-CRP levels were associated with a greater risk of stroke. The risk of development of stroke was significantly higher among males who are hypertensive having raised Hs-CRP levels in comparison to normotensive males with low serum Hs-CRP levels.
6	Liu et al (2014) [53]	Prospective Cross-sectional	This large population study concluded that CRP can be used as a screening tool to identify individuals with higher risk of ischemic stroke in Chinese population as they observed that higher Hs-CRP levels were positively associated with the risk of ischemic stroke, but not of intracranial hemorrhage and subarachnoid hemorrhage.

The Metabolic Syndrome and Diabetes Mellitus

Type 2 diabetes mellitus is one of the common chronic lifestyle diseases, which is characterized by the development of insulin resistance, and raised blood sugars. Type 2 diabetic patients develop microvascular and macrovascular complications. Macrovascular complications are mainly due to accelerated atherosclerosis, which is exacerbated by vascular inflammation and free radical injury contributed by a high glycemic state. Several markers of inflammation have been investigated with potential atherosclerotic risk. Among these markers of inflammation, the most widely used is the measurement of C-reactive protein and ESR. The other markers of inflammation are serum ferritin, serum albumin, and serum fibrinogen. Among these inflammatory markers, CRP has been found to be strong and independent for both the development of incident diabetes and cardiovascular complications [54].

Given that CRP actively contributes to the development of atherosclerotic plaque, instability in blood flow, and subsequent clot formation, it is also considered a cardiovascular disease risk factor. Most of the studies of CRP performed in the past in healthy populations are almost mostly limited to men. In women's health studies it is found that median levels of CRP are higher overall than levels observed in previous studies of men. Among apparently healthy middle-aged women baseline CRP concentration is an independent risk factor for cardiovascular disease. Therefore, CRP helps to predict vascular events even among low-risk subgroups of apparently healthy women with no other detectable apparent markers for disease [55].

The mechanism by which sustained chronic inflammation occurs is unclear. However, several vascular mediators like IL-1, IL-6, and TNF alpha are supposed to have a major role in chronic inflammatory processes. In that way, Hs-CRP is more sensitive to vascular inflammation. Since diabetes is also associated with atherosclerosis and chronic inflammation, we strive to find out the relationship associated between Hs-CRP levels and diabetes. Recently new reference standard for the use of Hs-CRP highly sensitive CRP assays was established by the World health organization, which is valid internationally. This has enhanced the usefulness of Hs-CRP as it is used widely as an ideal biomarker for the prediction of global cardiovascular risk. Raised levels of Hs-CRP are found to be associated with a higher risk of type 2 diabetes mellitus in patients suffering from metabolic syndrome [56].

On the basis of Hs-CRP levels, primary prevention can be initiated to prevent coronary heart disease events, reduce CHD deaths and decrease mortality and morbidity in diabetic patients. There is a direct co-relationship between glycosylated hemoglobin levels and Hs-CRP. Better control of blood sugars results in decreased Hs-CRP levels; therefore, it can be used as a prognostic marker in diabetic patients. This importance is highlighted by The UK Prospective Diabetes Study (UKPDS) trial [57], the atherosclerotic cardiovascular disease (ASCVD) risk score [58], and the justification for the use of statins in primary prevention: an intervention trial evaluating rosuvastatin (Justification for the Use of Statins in Prevention: an Intervention Trial Evaluating Rosuvastatin; JUPITER) [59]. All these studies have pointed out important relationships in the pathogenesis of atherosclerosis and Hs-CRP and have also highlighted the importance of good control of diabetes to reduce Hs-CRP levels as well as diabetes-related complications [60]. Intermediate hyperglycemia is known as a pre-diabetes state. Though it is a benign condition, it is associated with low-grade inflammation which results in the release of inflammatory markers. As Hs-CRP is a sensitive inflammatory marker, it helps to detect pre-diabetes. It is a good prognostic marker and this helps to predict cardiovascular complications in otherwise healthy-looking patients with raised blood glucose levels [61]. 

Metabolically healthy obese individuals are persons with a BMI more than or equal to 30 kg/m² without features of metabolic derangement like abnormal lipid profile, altered blood sugars, or metabolic syndrome. It is a state of low-grade inflammation. These individuals are at increased risk of developing type 2 diabetes mellitus and adverse cardiovascular events. Hs-CRP acts as a subclinical cardiovascular risk marker in these individuals (Table 4) [62]. 

**Table 4:** Results of various studies showing the role of Hs-CRP in diabetes mellitus. HbA1c: Hemoglobin A1C, Hs-CRP: High-sensitivity C-reactive protein.

Sr. No	Author (Reference)	Type of study	Conclusion
1	Ghule et al (2021) [61]	Cross- sectional	Hs-CRP can be used as an early and definite predictor of inflammation in pre-diabetes. It can be a marker of underlying deranged sugar levels and lipid profile in pre-diabetics as Hs-CRP levels were directly proportional to the cholesterol and low-density lipoprotein levels in this study.
2	Gupta et al (2020) [63]	Case-control	Hs-CRP levels were high in type 2 diabetes mellitus with higher risk levels associated with macrovascular complications compared to microvascular complications. The levels of Hs-CRP correlated with the duration of diabetes and HbA1c.
3	Sinha et al (2019) [64]	Cohort	Raised levels of serum Hs-CRP were associated with an increased incidence of diabetic neuropathy during a median follow-up of 7.8 years.
4	Pfützner et al (2006) [65]	Case-control	Hs-CRP is accepted as a predictive laboratory marker for cardiovascular disease risk in patients with type 2 diabetes mellitus.

Cancer

Cancers with chronic inflammation show raised CRP levels. It was shown in a population-based study that apparently healthy people with raised CRP levels have an association with an increased risk of development of carcinoma like carcinoma of lung cancer [66]. The results of epidemiologic studies revealed that elevated levels of CRP are indicators of poor prognosis and greater risk of early death compared with their counterparts with normal CRP levels or less elevated levels. For instance, studies show that the risk of colon cancer is less with a low grade of inflammation, which also shows raised CRP; therefore, various anti-inflammatory drugs may act as a novel therapy to reduce the incidence of colon cancer [44,67].

Obstructive Sleep Apnea (OSA)

Obstructive sleep apnea also has raised CRP levels. In patients suffering from OSA, significantly higher levels of IL-6 were found, and raised levels are found to be associated with the severity of the apnea-hypopnea index score. To increase the efficacy of continuous positive airway pressure ventilation and for effective treatment, levels of CRP and IL-6 can be monitored [68,69].

Rheumatoid Arthritis (RA)

CRP levels are also increased in rheumatoid arthritis. Disease activity score (DAS28), a tool required to calculate disease activity in newly diagnosed rheumatoid arthritis patients, assesses serum CRP levels along with 28 specified joints and a visual analog scale for global function. Thus Hs-CRP helps in assessing the severity of the disease. Similarly, a change in DAS28 score after treatment also helps in clinical decision-making in treating rheumatoid arthritis [70].

Age-Related Macular Degeneration (AMD)

Chronic inflammation in the eyes may accelerate age-related macular degeneration. It is a very common cause of blindness in the elderly population worldwide and patients present with progressive, painless loss of vision. This acquired disease develops due to degeneration of the pigmentary photoreceptor-retinal epithelial cell layer, Bruch's membrane, and chorio-capillaries complex [71,72]. Raised levels of CRP are found to have an association with an increased risk of development of AMD. It indicates choroidal vascular dysfunction and complement activation in AMD, which causes subsequent tissue damage. CRP plays a crucial role in the pathophysiology of AMD and is also useful in the assessment of the severity of AMD [73].

Alzheimer’s Disease

CRP levels are also associated with degenerative disorders of the nervous system like Alzheimer’s disease. It has features like gradually progressive dementia, the decline in cognitive functions of the brain, and other functional impairments. Chronic inflammation of the nervous system tissue plays a vital role in the pathogenesis of Alzheimer’s disease [74]. In Alzheimer's disease, senile plaque formation is an acute phase inflammatory state which is associated with high CRP levels. In our clinical practice, serum CRP levels have diagnostic value for mild and moderate Alzheimer's disease, as its levels are decreased among patients with a mild and moderate form of the disease. It is proven that decreased levels of CRP are linked with rapid functional and cognitive deterioration [75], whereas raised CRP levels indicate more risk of Alzheimer's disease development [76].

Parkinson's Disease

Parkinson's disease, a degenerative disorder of the nervous system, is characterized by decreased dopamine levels due to apoptosis of dopaminergic neurons in substantia nigra, and also the presence of Lewy bodies [77]. The neuroinflammatory process in Parkinson's disease is associated with elevated Hs-CRP levels [78] therefore, raised levels of CRP are found to be associated with a higher risk of development of Parkinson's disease. It has prognostic value in Parkinson's disease [79].

Fibrosis and inflammation

Collagen vascular diseases like dermatomyositis, polymyositis, and scleroderma show little CRP response. CRP levels are elevated in systemic lupus erythematosus (SLE) if there is the presence of serositis or synovitis. CRP levels are also raised in kidney failure. Patients with raised high CRP levels are at risk of developing severe peripheral vascular disease and inflammatory bowel diseases like ulcerative colitis and Crohn's disease [80].

Interventions to reduce CRP levels

Lifestyle Interventions

Exercise has a positive effect on serum CRP levels as it causes a decrease in CRP. In a few studies, it was found that increased CRP levels or dyslipidemia before beginning exercise were associated with greater reductions in CRP [81]. In past interventional studies, smoking cessation, exercise, and weight reduction interventions were shown to lower CRP levels among study participants who are at high risk for the development of non-communicable diseases like diabetes mellitus, cerebrovascular events, and ischemic cardiovascular disease (Table 5) [82,83].

**Table 5:** Effective interventions to lower Hs-CRP levels. Hs-CRP: High-sensitivity C-reactive protein

Interventions	Examples of Interventions
Lifestyle modifications	Exercise, optimal blood pressure control, moderate alcohol consumption, smoking cessation, and weight control
Pharmacological management	Statins, aspirin, fibrates, niacin, thiazolidinediones
Dietary and nutrition supplementation	Diets containing green leafy vegetables, fruits, fatty fish, and nuts. Diet rich in curcumin, fenugreek, ginger, green tea polyphenol, Isoflavones, L-carnitine, magnesium, probiotics, omega-3 fatty acids, zinc, Vitamin- C, D, and E.

Statin Therapy

Lipid-lowering drugs like statins (HMG-CoA reductase inhibitors) could be beneficial in the treatment of raised levels of CRP. This result is based on the findings of the JUPITER trial that found that elevated CRP levels without hyperlipidemia benefited from rosuvastatin [59]. Various studies have concluded that statins reduce Hs-CRP levels. Due to their anti-inflammatory and lipid-lowering effects, they are the drug of choice in the management of hypercholesterolemia among patients with high Hs-CRP levels [84,85]. Antidiabetic drugs (metformin, thiazolidinediones, insulin), angiotensin-receptor blockers, and statin combined with ezetimibe combination therapy have been shown to lower Hs-CRP levels [86,87].

Strengths and limitations

The topic of this literature review article is very broad and we have included observational studies, clinical trials, systemic reviews, and meta-analyses. The majority of the selected studies have explained the effect of Hs-CRP on various non-communicable diseases. However, a few of them have also explained the biological functions of CRP and its role in the pathogenesis of non-communicable diseases. Other limitations of this study are that we could not include studies from Cochrane and Embase electronic databases and excluded non-English literature.

## Conclusions

CRP is a nonspecific marker of inflammation and has an important value in the management of various inflammatory or immunomodulatory states, including microbial infections due to bacteria, trauma, tissue damage, and neuro-degenerative condition during the acute phase as well as during recovery. An increase in serum CRP levels occurs during both acute inflammation and chronic inflammation. Results of various experimental and clinical studies conducted in the past showed a strong association between raised serum CRP levels with more risk of development of ischemic heart disease, type 2 diabetes mellitus, neurodegenerative diseases like Parkinsonism, Alzheimer’s disease, hemorrhagic as well as ischemic stroke, autoimmune diseases like systemic sclerosis, rheumatoid arthritis, and systemic lupus erythematosus. Hs-CRP is a marker of chronic inflammatory conditions, which is readily available and affordable, and it also gives an idea about the disease state. Better knowledge and understanding of the synthesis of Hs-CRP during the inflammatory process, its activation, detailed mechanism, and dissociation in the body during disease is important. This understanding will help to plan a treatment strategy that will minimize tissue damage, improve the outcome of various non-communicable, chronic inflammatory disorders, and improve patient management.
